# The Final Overthrow of Koch’s Theory Respecting the Non-Communicability of Bovine Tuberculosis to Human Beings

**Published:** 1902-09

**Authors:** 


### EDITORIALS.

### THE FINAL OVERTHROW OF KOCH'S THEORY RESPECTING THE NON-COMMUNICABILITY OF BOVINE TUBERCULOSIS TO HUMAN BEINGS.

KOCH'S labored effort to prove that bovine tuberculosis cannot be communicated to human beings was practically wholly based upon the experiments from which he claimed to have demonstrated that human tuberculosis could not be communicated to animals of the bovine species. As has been so often pointed out, the facts which he presented were not conclusive, if proved; for it might easily be possible that bovine tuberculosis could be communicated to human beings, even though human tuberculosis could not be communicated to calves; but Arloing, a recognized authority, has come forward (*Bulletin de l'Academie de Medecine and Presse Medicale*) with a carefully studied series of observations which show conclusively that human tuberculosis may be communicated to various species of lower animals. In his experiments he produced tuberculosis in twenty-three animals, all of which were pronounced by Koch to be non-tubercularizable by infection from human beings. Of these animals, there were three calves, six sheep, ten goats, and three donkeys. Arloing further shows by careful analysis of the observations of Koch that seven out of thirty-four animals inoculated by him with human virus were actually infected. Every sanitarian will be glad to welcome this thoroughgoing exposure of Koch's most ill-advised and mischievous announcement.—*Modern Medicine*, July, 1902.

